# The extensive use of 3D printing in trauma does not yet fit the value-based healthcare era

**DOI:** 10.1186/s10195-024-00789-9

**Published:** 2024-10-12

**Authors:** Andrea Fidanza, Gianfilippo Caggiari, Alessio Giannetti, Manuel G. Mazzoleni

**Affiliations:** 1 Orthopaedic Mininvasive and Computer Assisted Surgery Unit, Dep. of Life, Health and Environmental Sciences-University of L’Aquila, Piazzale S.Tommasi, 1, 67100 L’Aquila, Italy; 2 Orthopaedic and Traumatology Department, Sassari University Hospital, Sassari, Italy; 3 Orthopaedic and Traumatology Unit, “SS Maria dello Splendore” Hospital, Giulianova, Italy; 4 Hip Department (CAD), Gaetano Pini-CTO Orthopaedic Institute, Milan, Italy

Dear Editor,

We are very pleased to read the comment by Li R. et al. “*Are the costs of 3D printing for surgical procedures yet to be definitively assessed?*” [1] on one of our recent studies [2].

Three-dimensional (3D) printing in medicine has generated significant enthusiasm in recent decades [3, 4]. However, the expert knowledge and substantial financial investment required to apply these technologies are hard to quantify, especially in traumatology. The variety of fractures and the urgency of trauma care make sophisticated cost assessment challenging, unlike in elective prosthetic surgery [5].

This uncertainty and the lack of definitive answers give rise to new questions.

The authors of the commentary letter cited Chen et al., who compared outcomes of patients treated for proximal humeral fractures (PHF) using traditional preoperative planning during the first 4 years of retrospective data collection and thereafter versus augmented reality and life-size 3D printing of the fractures [6]. Conversely to Chen et al., in our study the neck–shaft angle and the loss of humeral head height have not been calculated because postoperative computed tomography (CT) scans were not performed, where these parameters have been validated. In our experience, the reduction of the fracture was considered acceptable when the fluoroscopy image during the surgery was superimposable onto the 3D model previously reduced. The postoperative constant score (CS) did not reveal any significant differences between the two reports. Both sets of results fall within the standard deviation highlighted in a recent meta-analysis on PHF treated with plate and screws, where the CS was reported as 75 ± 15.8 points [7].

However, the situation changes when general quality of life assessment measures, such as the Short Form 36 Health Survey Questionnaire (SF-36) or [8], are employed. While these tools are valuable and routinely used for each of our patient, we acknowledge, that when reporting specific outcomes for trauma, these scales may introduce biases due to the influence of co-existing or subsequent pathologies, as well as the inability to administer a preoperative assessment.

The most frequently questioned aspects of new technologies are their cost and time commitment, which are largely determined by how expenses are interpreted [5] and how 3D prints are produced. Since Chen et al. developed the computer platform themselves, they did not report any additional costs associated with virtual reality. However, if this technology were to be commercialized, there would likely be expenses related to software purchase, hardware-dependent updates, and possibly even for training on different bones or in other medical fields. By adopting a straightforward approach to allocate spending between direct and indirect costs, we have achieved savings primarily through the reduced use of active operating room time and, consequently, all associated personnel who would otherwise be unable to create value elsewhere. Naturally, an initial investment is required for the purchase of a 3D printer and computer, but the software we used is available free of charge for non-commercial purposes (InVesalius software, MCTI, Brazil). Additionally, by applying the well-established engineering protocol previously published [9], this method can be replicated in other settings, and once post-production skills are acquired, it can be used on other anatomical areas without incurring additional costs or requiring updates. In fact, it is now standard practice in several Italian residency program to use 3D-printed models for other joint fractures, such as those of the wrist, heel, and tibia [10]. Unlike virtual simulation, this approach allows for hands-on surgical practice for young surgeons, thereby enhancing their safety in performing complex surgeries and accelerating the improvement of clinical outcomes described earlier. Furthermore, the 3D printing material we chose (polylactic acid filament filled by expanded polyurethane foam to simulate spongy bone) is an eco-friendly, non-petroleum-based materials that can be sterilized, allowing the surgeon to handle it in the operating room to visually confirm the planned intervention.

In conclusion, we believe that 3D printing can be a valuable tool for enhancing preoperative planning compared with traditional techniques and for providing superior training opportunities for young surgeons compared with online software. However, it is important to note that this technology has not yet demonstrated significant improvements in clinical outcomes. Therefore, we agree with the commentors that, in the context of value-based healthcare, the presence of a 3D printer in every medical center is not essential. Nevertheless, its use can be particularly beneficial in research and training institutions, as well as in managing the most complex cases.

## Data Availability

Not applicable.
